# Global multi‐omics analysis of brain‐targeting exerkines in progressive treadmill‐exercised rodents

**DOI:** 10.1002/alz70855_101454

**Published:** 2025-12-23

**Authors:** Hash Brown Taha

**Affiliations:** ^1^ Keck School of Medicine, University of Southern California, Los Angeles, CA, USA

## Abstract

**Background:**

Exercise offers neuroprotective and cognitive benefits against neurodegeneration in Alzheimer's disease, hypothesized to be due to the secretion of signaling molecules known as brain‐targeting exerkines. However, the responsiveness of these exerkines across multiple organs remains poorly understood.

**Method:**

We leveraged the publicly available (MoTrPAC) dataset. Rats aged 6 months underwent a progressive treadmill training protocol. Blood (cells and plasma) and 18 solid tissues such as brain (cortex, hippocampus, hypothalamus) were collected, and multi‐omics analyses were performed on them. We examined the protein and transcriptional expression of several brain‐targeting exerkines across all collected tissues.

**Result:**

Brain‐targeting, tissue‐specific exerkine responsiveness ranked as follows: prosaposin > cathepsin B > Clusterin = FNDC5/irisin > platelet factor 4 > brain‐derived neurotrophic factor (BDNF) = Gpld1. However, both the protein and transcriptional expression of all these brain‐targeting exerkines remained unchanged in the brain across males and females throughout all 8 weeks of treadmill training.

**Conclusion:**

Contrary to previous studies, our analysis shows that brain‐targeting exerkines remained unchanged in the brain after exercise, challenging the notion of a body‐brain exerkine axis and questioning its role in neuroprotection and cognitive benefits.

